# Transcriptome analysis of granulosa cells after conventional vs long FSH-induced superstimulation in cattle

**DOI:** 10.1186/s12864-018-4642-9

**Published:** 2018-04-16

**Authors:** F. C. F. Dias, M. I. R. Khan, M. A. Sirard, G. P. Adams, J. Singh

**Affiliations:** 1 Department of Veterinary Biomedical Sciences, Western College of Veterinary Medicine, University of Saskatchewan, 52 Campus Drive, Saskatoon, SK S7N 5B4 Canada; 2 Departement des Sciences Animales, Centre de Recherche en Biologie de la Reproduction, Universite’ Laval, Sainte-Foy, Quebec, G1K 7P4 Canada; 3 Department of Theriogenology, University of Veterinary and Animal Sciences, Lahore, 54000 Pakistan

**Keywords:** Cattle, Follicular waves, Follicle growth, Granulosa cells, Gene expression, Genomic analysis, Microarrays, Ovary, Superstimulation

## Abstract

**Background:**

Prolongation of superstimulatory treatment appears to be associated with a greater superovulatory response and with greater oocyte maturation in cattle. A genome-wide bovine oligo-microarray was used to compare the gene expression of granulosa cells collected from ovarian follicles after differing durations of the growing phase induced by exogenous FSH treatment. Cows were given a conventional (4-day) or long (7-day) superstimulatory treatment (25 mg FSH im at 12-h intervals; *n* = 6 per group), followed by prostaglandin treatment with last FSH and LH treatment 24 h later. Granulosa cells were harvested 24 h after LH treatment.

**Results:**

The expression of 416 genes was down-regulated and 615 genes was up-regulated in the long FSH group compared to the conventional FSH group. Quantification by RT-PCR of 7 genes (*NTS, PTGS2, PTX3, RGS2, INHBA, CCND2* and *LRP8*) supported the microarrays data. Multigene bioinformatic analysis indicates that markers of fertility and follicle maturity were up-regulated in the long FSH group.

**Conclusion:**

Using the large gene expression dataset generated by the genomic analysis and our previous associated with the growth phase and gene expression changes post LH, we can conclude that a prolonged FSH-induced growing phase is associated with transcriptomic characteristics of greater follicular maturity and may therefore be more appropriate for optimizing the superovulatory response and developmental competence of oocytes in cattle.

## Background

An increase in peripheral FSH concentrations precedes emergence of each new follicular wave in cattle [1]. A follicular wave is characterized by the emergence of a growing cohort of FSH-responsive follicles, one of which is selected for continued growth (dominant follicle) while remaining subordinates follicles regress [2]. The majority of estrous cycles in cattle are composed of 2 or 3 follicular waves, the last of which culminates in ovulation of the dominant follicle. Major differences between 2- vs 3-wave cycles are a significantly shorter interovulatory interval (19 vs 23 days, respectively), a longer growing phase of the dominant follicle of the first wave (8 vs 7 days, respectively) and a longer growing phase of the final (ovulatory) wave (9 vs 6 days, respectively) [3]. During a 2-wave estrous cycle, the ovulatory follicle grows for 6 days under a high-progesterone environment, while that of a 3-wave cycle grows for only 3 days under a high-progesterone environment [4]. Thereafter, circulating progesterone concentrations are low for both 2- and 3-wave cycles, for approximately 3 days following luteolysis [4].

The period of growth and maximal size of the dominant follicle are inversely related to the circulating concentration of progesterone [5]. Dominant follicles maintained under a low-progesterone environment for extended periods become oversized, persistent follicles [5] containing an oocyte of decreased developmental competence [6–9]. However, the effect of different durations of elevated progesterone exposure during the growing phase of the ovulatory follicle (analogous to 2- or 3-waves cycle) is contradictory [10–13]. In more recent studies, extending the period of progesterone exposure during the growing and early-static phases of the ovulatory follicle by 3 days did not affect oocyte competence of the dominant follicle [14] or that of superstimulated follicles [15], but was associated with a greater superovulatory response [15, 16]. In addition, a longer growing phase of the ovulatory follicle during superstimulation was associated with greater nuclear and cytoplasmic maturation of oocytes [17].

During folliculogenesis, somatic cells and germ cells interact through complex paracrine signalling via heterologous gap junctions to coordinate nuclear and cytoplasmic maturation of the oocyte [18]. Granulosa cell signalling affects energy uptake by the oocyte [19, 20], oocyte growth and development [21], and transcriptomic activity of the oocyte [22]. Granulosa cell analysis is therefore a valuable method of assessing follicular and oocyte health status. The effect of ovarian superstimulation on bovine granulosa cells has been the focus of recent studies (reviewed in [23]), and results revealed that multiple follicles growing during a standard 4-day superstimulation protocol were not as mature as a single dominant follicle from a natural cycle [24]. Based on the findings that prolongation of superstimulatory treatment appears to be associated with a greater superovulatory response in cattle [25, 26], the influence of prolonged FSH treatment on granulosa cell function, and the corresponding effect on the contained oocyte, is of interest in guiding our understanding of granulosa-oocyte health. In this context, it becomes very interesting to study the effect of extending the superstimulation protocol by 3 days on the global gene expression of follicular cells.

Therefore, the objective of the study was to compare the transcriptomic profile of granulosa cells from ovarian follicles exposed to a short duration (conventional 4-day treatment) vs long duration (7-day treatment) of FSH-stimulated growth. Our hypothesis is that more follicles will advance in differentiation and this will improve LH responsiveness.

## Methods

### Animals and treatments

Twelve mature Hereford cross-bred beef cows, weighing 515 to 795 kg, and maintained in outdoor pens, were used. Procedures were conducted in accordance with the guidelines of the Canadian Council on Animal Care and were approved by University of Saskatchewan Protocol Review Committee.

A luteolytic dose of prostaglandin F_2α_ (PGF_2α_; 500 μg cloprostenol im; Estrumate, Schering-Plough Animal Health, Pointe-Claire, PQ, Canada) was given twice, 14 days apart to synchronize estrus. The ovaries were examined by transrectal ultrasonography every 24 h after the second PGF_2α_ treatment to detect ovulation. Five to 8 days after ovulation, follicles ≥ 5 mm in diameter were ablated by transvaginal ultrasound-guided follicle aspiration to synchronize the emergence of a new follicular wave 1 day later [27]. An intravaginal progesterone-releasing device (CIDR-B, 1.38 g progesterone, Pfizer Canada Inc., Kirkland QC, Canada) was placed in the vagina immediately after follicle ablation.

**Fig. 1:**
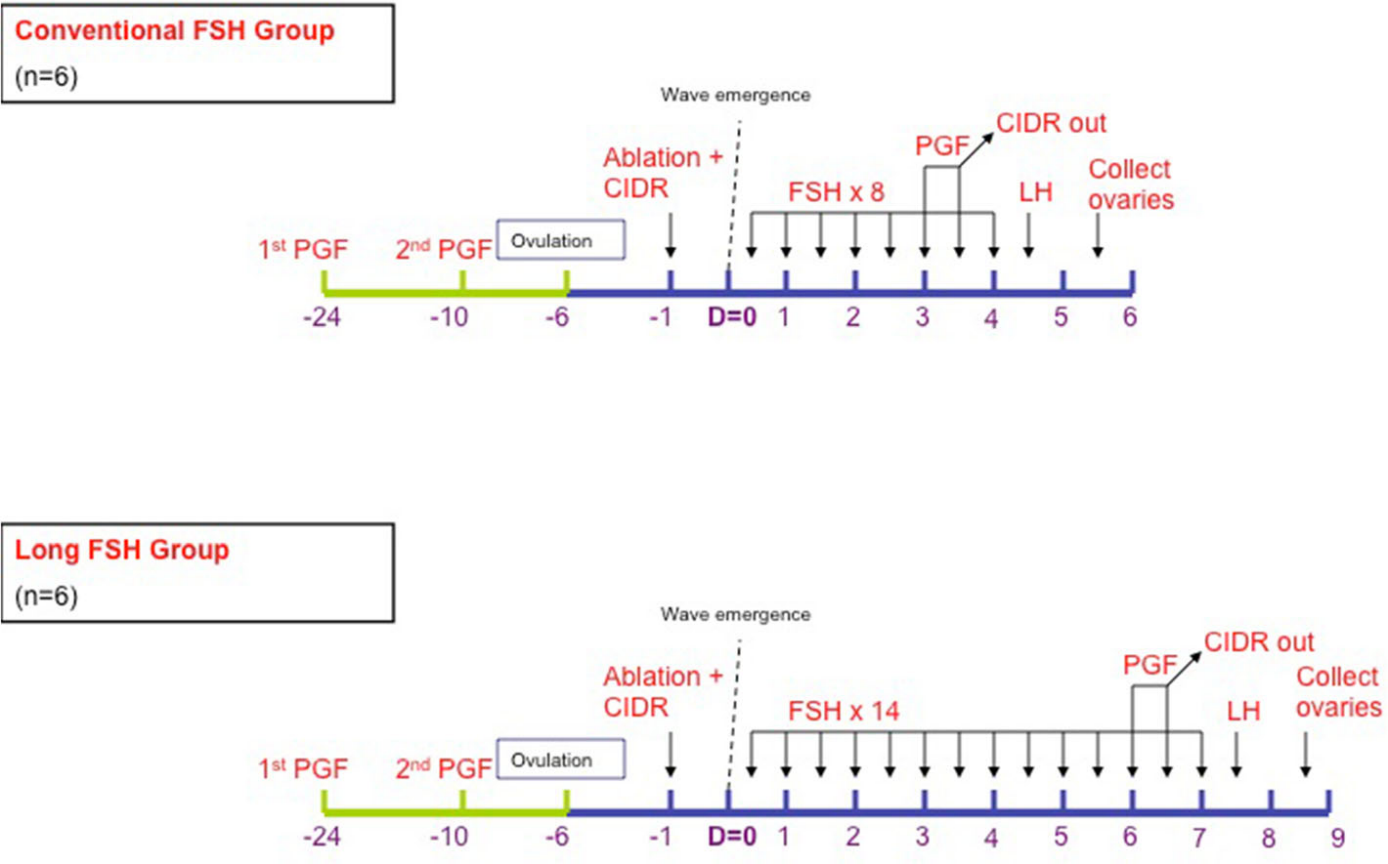
Experimental protocol used to test the effect of the duration of FSH superstimulation on gene expression of granulosa cells in cattle. Transvaginal ultrasound-guided follicle ablation was performed 5 to 8 days after ovulation to synchronize wave emergence (Day 0). Beginning on Day 0, FSH was given at 12-h intervals for 4 days (conventional FSH group) or 7 days (long FSH group; *n* = 6 cows per group). Cows were given PGF_2α_ twice on Day 3 in the conventional FSH group or Day 6 in the long FSH group. Cows were ovariectomized 24 h after LH treatment

The cows were allocated randomly to two groups (*n* = 6/group) and given either a 4-day (conventional FSH) or 7-day (long FSH) superstimulatory protocol (Fig. 1). Starting on the day of wave emergence (Day 0), i.e., 1 day after follicle ablation, cows in the conventional FSH group were given 8 im treatments of FSH (Folltropin-V; Bioniche Animal Health, Belleville ON, Canada; each equivalent to 25 mg of NIH-FSH-P1) at 12-h intervals over 4 days. Cows in the long FSH group were given 14 im treatments of FSH over 7 d (each dose equivalent to 25 mg of NIH-FSH-P1). Cows in the conventional FSH group were given two luteolytic doses of PGF_2α_ im 12 h apart on Day 3, whereas the cows in the long FSH group were given the same PGF_2α_ treatment on Day 6. The CIDR was removed concurrent with the second prostaglandin treatment. Cows were treated with 25 mg LH im (Lutropin-V, Bioniche Animal Health) 24 h after CIDR removal. Cows were ovariectomized 24 h after LH treatment.

### Tissue collection

Bilateral ovariectomy was performed by colpotomy, as described [28]. Briefly, caudal epidural anesthesia was induced with 2% lidocaine HCl and 0.01 mg/ml of epinephrine (Bimeda-MTC Animal Health Inc., Lavaltrie, QC, Canada). The perineum was washed using an iodine-based detergent and solution. A small incision was made in the dorso-lateral aspect of the vaginal fornix using a scalpel blade; the peritoneum was manually punctured to allow access to the peritoneal cavity. The mesovarium was compressed with a lidocaine/epinephrine-soaked gauze and a plastic clip was placed across the ovarian pedicle to compress the ovarian vessels. The chain of an ecraseur (19” Chassaignac; German-made; Jorgensen Lab, Loveland, Colorado, USA) was looped around the ovary and slowly tightened until the ovarian pedicle was severed. The ovaries were placed in a polyethylene bag on ice and transported to the laboratory within 5 min after collection. After ovariectomy number of follicles ≥ 5 mm was counted and the three largest follicles (in the pair of ovaries) were selected. The goal was to include both antral and mural granulosa cells in the sample for analysis. Follicular contents were aspirated using a 20 gauge needle and a syringe, and the follicle was flushed 3 times with Dulbecco’s phosphate buffer saline (dPBS, Invitrogen Corporation, catalog 14,190–144, Burlington, ON, Canada). The cumulus-oocyte-complex was identified by stereomicroscopy and separated from the follicular aspirate, and the remainder of the follicular aspirate and the saline flush was centrifuged to collect antral granulosa cells. The collapsed follicle was then opened in half using a scalpel blade. Identification of the 3 largest follicles was confirmed using a ruler to measure follicle diameter. The inside of the follicular wall was scraped with a microbiology culture-loop (LightLabs, catalog # PD104, Dallas, TX, USA) to remove the mural layer of granulosa cells, which were then placed together with the antral granulosa cells. The granulosa sample (mural and antral) of each follicle was placed in an Eppendorf tube (sterile, RNAse- and DNAse-free) and plunged into liquid nitrogen and stored at -80 °C for microarray and RT-PCR analyses. The follicular fluid was stored at -80 °C for radioimmunoassay of progesterone and estrogen concentrations.

### RNA extraction and amplification

Total RNA was extracted using the Trizol extraction method, according to the manufacturer’s instructions (Invitrogen Life Technology; Thermo Fisher Scientific, Waltham, Massachusetts, USA), and resuspended in 50 μl of nuclease-free water. The RNA was purified using the Arcturus *PicoPure RNA* Isolation and purification Kit (Catalog KIT0204, Applied Biosystem, Concord, Ontario, Canada) following the manufacturer’s protocol. The purification process included DNAse treatment to remove genomic DNA, and final purified RNA was recovered in 15 μl of elution buffer. RNA quality was evaluated using a Bioanalyzer-2100 (Agilent Technologies Inc., Palo Alto, CA, USA) with the RNA NanoLab Chip (Catalog # 5067–1511; Agilent; Santa Clara, California, USA). RNA samples with an RNA integrity number (RIN) greater than 5 were used for microarray hybridizations. A linear amplification process was chosen with the intent of increasing the amount of RNA for microarray hybridizations. Equal amounts of RNA from the three largest follicles were pooled and a total of 5 ng of RNA from the pooled sample was used for RNA amplification. Linear amplification was performed using two 6-h rounds of T7 RNA polymerase (RiboAmp HS^Plus^ RNA Amplification Kit; Molecular Devices, Sunnyvale, CA, USA), following manufacturer’s directions, and the amount of antisense RNA (aRNA) produced was measured using NanoDrop ND-1000 (NanoDrop Technologies, Wilmington, DE, USA).

### Sample labeling, hybridization and microarray scanning

For each sample, 2.5 μg of aRNA were labelled using DY-547/647 (Red - CY5 and Green CY3) fluorescent dyes from ULS Labelling Kit (EA-006, Kreatech Diagnostics, Amsterdam, The Netherlands) according to the manufacturer’s protocol. With the intent of removing any non-reacted ULS-label material, another round of aRNA purification was performed using the Pico-Pure RNA Isolation Kit but without DNAse I treatment. Pure labeled aRNA was eluted with 11 μl elution buffer. Labelling efficiency was measured using the NanoDrop ND-1000. A minimum of 30 pmol/μg of labeling signal was required to proceed with hybridization. A hybridization mix was prepared using: 825 ng each of cyanine (Cy3 and Cy5) labeled amplified aRNA; Agilent and tomato spikes; nuclease free water; 10× blocking agent; and a 25× fragmentation buffer, n a total volume of 55 μl. The hybridization mix was then pipetted into the hybridization slides. Three biological replicates in each group (conventional vs long FSH) were used in the experimental design, in a dye-swap set up. Overall, 6 hybridizations were performed using a custom-built bovine oligo-microarray slide (EmbryoGENE EMBV3 manufactured by Agilent; Design ID: 028298, GEO accession # GPL13226). The slide contained 45,220 oligo-nucleotide probes. Each gene had a duplicate oligo-nucleotide probe and the slide also included Agilent’s positive and negative controls (4 arrays per slide; 44 K probes per array). Oligo-nucleotide sequences were taken from Oligo Microarray Consortium database (BOMC, http://www.bovineoligo.org).

Hybridization were performed (Agilent Technologies Inc., Palo Alto, CA, USA) using 2× GEx hybridization buffer HI-RPM, at 65 °C in a preheated oven for 17 h with a rotator speed of 10 rpm. Slides were washed with two buffers from the Gene Expression (GE) wash buffer kit (Agilent Technologies Inc., catalogue # 5188–5327), according to manufacturer’s protocol. Slides were then dipped in 100% acetonitrile for 10 s at room temperature and washed with stabilization and drying solution for 30 s at room temperature. The slides were scanned immediately using a Power scanner (Tecan US Inc., Durham, NC, USA). After image acquisition, scanned images were analyzed and quantified using Array-Pro Analyzer software (Media Cybernetics, Silver Spring, MD, USA).

### Data normalization and statistical analyses

Raw signal intensity files were uploaded to the EmbryoGENE Laboratory Information Management System (LIMS) and microarray analysis platform (ELMA). Quality control was evaluated using Gydle software (http://www.gydle.com/). Signal intensity files were analyzed using FlexArray software (version 1.6.1; [29]. Simple background subtraction was done with the FlexArray software using the median foreground intensity files. If background intensity was higher than the obtained foreground intensity, negative values were replaced with 0.5 as a default. Data were normalized within and between arrays using the Loess and Quantile normalization methods, respectively. Linear Models for Microarrays (Limma) was performed to obtain differentially expressed genes in the long FSH group relative to the conventional FSH group [30, 31] using a fold-change of ≥ 2 and a *P*-value of ≤0.05 as a threshold. The false discovery rate (FDR) was determined using the Benjimeni-Hocheberg method to narrow down the true positive genes. A fold-change of ≥ 2 and *P* value of ≤ 0.05 was also used for FDR. Data were deposited in NCBI Gene Expression Omnibus 226 (www.ncbi.nlm.nih.gov/geo/) with a GEO series accession number; GSE80289. A *p*-value of ≤0.1 was taken as statistically significant for RT-PCR data.

### Functional annotation and pathway analysis

The list of differentially expressed gene generated by Limma analysis was uploaded into Ingenuity Pathways Analysis (IPA Version: 14400082; Ingenuity Systems, www.ingenuity.com) to identify gene networks. Gene networks were used to identify likely biological functions, molecular processes and disorders, and most related pathways. IPA analyses are based on data from human and mouse studies.

### Real-time PCR (RT-PCR)

Seven genes (*NTS, PTGS2, PTX3, RGS2, INHBA, CCND2* and *LRP8*) were selected for validation using RT-PCR. Primers were designed using Primer3 (v.0.4.0, http://frodo.wi.mit.edu/primer3/), analyzed using IDT PrimerQuest tool (Oligo Analyzer, http://scitools.idtdna.com/analyzer/Applications/OligoAnalyzer/; https://www.idtdna.com/calc/analyzer), and blasted using NCBI (http://www.ncbi.nlm.nih.gov/tools/primer-blast/index.cgi?LINK_LOC=BlastHome; http://blast.ncbi.nlm.nih.gov/Blast.cgi?PROGRAM=blastn&BLAST_PROGRAMS=megaBlast&PAGE_TYPE=BlastSearch&SHOW_DEFAULTS=on&LINK_LOC=blasthome). Selected primers met the following criteria: 20 to 24 base pairs, 55° to 65 °C melting temperature, 40% to 60% of CG content, no hairpin, self-dimer or hetero-dimer formation, and specific to the gene of interest. A list of the primers selected is presented on Table 1.

**Table 1:** Primers used for RT-PCR

Genes	Strand	Primer sequence	Annealing temperature (°C)
	Forward	5’-CATGAGATGGCAGTCAATTTGT-3’	53.6
EIF2B2	Reverse	5’-CTTGAACATAGGAGCACAGACG-3’	55.5
	Forward	5’-TGTGTCCCTCTTGCTGAGTTT-3’	56.4
SF3A1	Reverse	5’-ATTCCTGGTTTCACGTCTCCTA-3’	55.5
	Forward	5’-TGGACTCAGAAGTATGCGATGT-3’	55.8
UBE2D2	Reverse	5’-CTTCTCTGCTAGGAGGCAATGT-3’	56.6
	Forward	5’-AGTGTTCCCTCTTGGAAAATGA-3’	60
NTS	Reverse	5’-TCTTCCTGAATCAACTCCCAGT-3’	60.1
	Forward	5’-AAAGCTCTAGGGGGTTCTCGT-3’	56.3
PTGS2	Reverse	5’-TGTCAGCACATCCAGGGTAA-3’	56
	Forward	5’-GGCAGACTCACAGGCTTCAATATC-3’	57.6
PTX3	Reverse	5‘-CCTTCTCCAGTCTCCCTTTCAACT-3’	58.2
	Forward	5’-AAAGCCGCAGATCACCACAGAA-3’	59.3
RGS2	Reverse	5’-TCCAGCTTGAGACACACCACAT-3’	58.6
	Forward	5’-CGACTTCATCGAACACATCCTTCG-3’	57.7
CCND2	Reverse	5’-CTATTCAGCAGCACCACCTCAATC-3’	57.8
	Forward	5’-ACGCAAAGTTCTCGCAAGCTCA-3’	59.7
LRP8	Reverse	5’-TGCC ATTTCCTCCTCAAACAGG-3’	57.6
	Forward	5’-CCAAAGGATGTACCCAACTCTC-3’	59.8
INHBA	Reverse	5’-GTCCGATGTCGTCCTCTATCTC-3’	60.7

Primers were tested by performing RT-PCR of cDNA from pooled granulosa cell samples. After cDNA was amplified during the PCR reaction (see below), the amplicon was run by electrophoresis on a standard 1% agarose gel to determine the size of the band. The band was cut and eluted using a QIAquick Gel Extraction kit (Cat# 28704, Qiagen, Toronto, ON, Canada), quantified using NanoDrop ND-100 and sequenced (3 × ABI 3730xl Sanger sequencing). Primers were only used if the sequence matched the desired amplicon. The amplicon was also used for generating a standard curve. The standard curve went from 10^− 2^ to 10^− 11^ ng/μl. Real time PCR was performed on a Stratagene Mx3005P fast thermal cycler (Applied Bioscience, Concord, Ontario, Canada) using SYBR Green master mix (Applied Bioscience). Cycle threshold (Ct) was recorded, and the expression of the gene of interest was normalized to the geometric mean of UBE2D2, EIF2B2 and SF3A1 using the Relative Expression Software Tool [32].

### Radioimmunoassays

Follicular fluid concentrations of estradiol and progesterone were measured by radioimmunoassay. Slaughterhouse ovaries were used to obtain a charcoal-extracted pool of fluid from follicles 3 to 8 mm in diameter, which was used to prepare the standards and dilute follicular fluid samples. The standard curve ranged from 5 to 1000 pg/ml for estradiol and 0.1 to 40 ng/ml for progesterone. Samples were diluted using the charcoal-extracted pooled follicular fluid so that hormone concentrations fell within the limits of the standard curve and samples were assayed in duplicates. Estradiol was measured with a modified human double-antibody radioimmunoassay kit (Catalog # KE2D1, Coat-A-Count; Siemens Healthcare Diagnostics Inc.; Mississauga, ON, Canada), using dilutions that ranged from 1:25 to 1:500. Estradiol was measured in two assays; the intra-assay coefficient of variation was 11% and the inter-assay coefficient of variation was 8%. Progesterone was measured using a commercial radioimmunoassay kit (Catalog # TKOP1, Coat-A-Count; Siemens Healthcare Diagnostics Inc.; Mississauga, ON, Canada) and all samples were diluted 1:10 and measured in a single assay. The intra-assay coefficient of variation was 6%. Hormone data were compared between groups by student’s t-test.

## Results

**Fig. 2:**
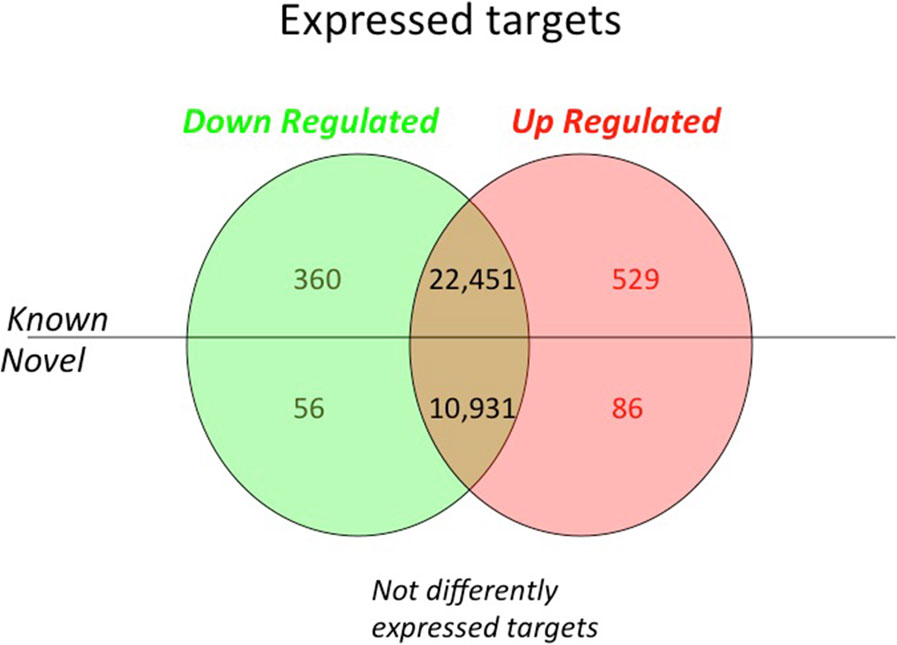
Venn diagram summarizing microarray analysis of bovine granulosa cells sampled from cows after a long (7-day) vs conventional (4-day) FSH superstimulatory treatment protocol. Cells were obtained from the FSH-stimulated follicles 24 h after exogenous LH treatment in both groups. Limma was used for statistical analysis and up- and down-regulated genes were identified as those expressing ≥ 2-fold-change with a *P* value of ≤ 0.05

A total of 1031 genes were differentially expressed in the long FSH group compared with the conventional FSH group (Fig. 2). Of the differentially expressed genes, 416 had lower expression (i.e., down regulated) and 615 had higher expression (i.e. up-regulated) in the long FSH group compared with the conventional FSH group (≥ 2-fold change; *P* value ≤ 0.05), and 142 were novel transcripts. The 10 most up- and down-regulated granulosa cell gene transcripts in the long FSH group compared to the conventional FSH group are listed in Table 2.

**Table 2:** The top 10 up- and down-regulated genes in bovine granulosa cells sampled from cows after a long (7-day) vs conventional (4-day) FSH superstimulatory treatment protocol (3 largest follicles pooled)

Genes	Description	*P* value	Fold change
Up-Regulated			
PTX3	Pentraxin 3	3.7 × 10^−13^	5.904
VNN3	Vanin 3	3.3 × 10^−11^	5.281
POSTN	Pcriostin, Osteoblast specific factor	1.4 × 10^−07^	4.241
PTGS2	Prostaglandin cndopcroxidc synthase 2	1.5 × 10^−07^	4.182
GRIA3	Glutamate receptor, ionotropic, AMPA3	8.4 × 10^− 07^	4.175
RGS2	Regulator of G protein signaling 2	2.9 × 10^−06^	3.702
GFRA1	GDNF family receptor alpha I	7.5 × 10^−05^	3.679
PLA2G4A	Phospholipase A2 group Iva (cytosolic, Ca dependent)	4.4 × 10^−07^	3.605
CRISPLD2	Cysteine rich secretory protein LCCL domain containing 2	2.9 × 10^− 05^	3.604
GIMAP4	GTPase IMAP family member 4	9.0 × 10^−12^	3.545
Down-Regulated			
INHBA	Inhibin beta A	2.0 × 10^−07^	−3.978
LRP8	Low density lipoprotein receptor-related protein 8	1.4 × 10^−07^	−3.784
CCND2	Cyclin D2	5.9 × 10^−09^	− 3.437
SRGN	Serglycin	1.2 × 10^−05^	−3.055
BEX2	Brain expressed X-linkcd 2	3.3 × 10^−07^	−3.055
INHBB	Inhibin beta B	2.9 × 10^−07^	−3.031

### Functional classification of transcripts

Among 1031 differentially expressed genes or isoforms, 889 were annotated for functional classification by Ingenuity Pathway analysis (IPA) software. Analysis of molecular (*P*-value range from 4.05 × 10^− 19^ to 2.77 × 10^− 04^) and cellular functions (P-value range from 2.12 × 10^− 04^ to 2.55 × 10^− 04^) revealed that those most affected by long FSH treatment were related to cellular growth and proliferation, cell death, cellular development, and cellular function and maintenance (based on number of genes in the function). The most affected disease categories were cancer, genetic disorders, tissue development, and reproductive system disease.

### Network analysis

**Fig. 3:**
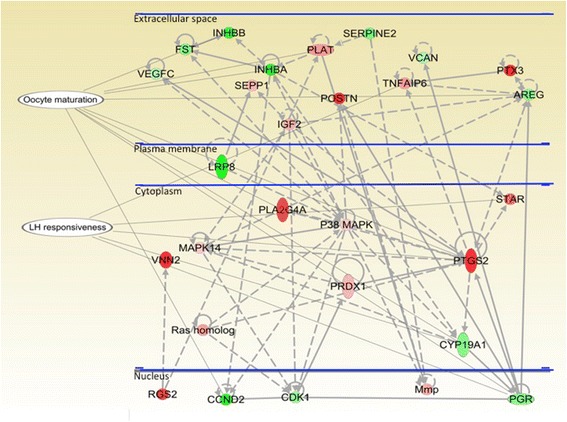
Network of up- (red) or down-(green) regulated genes and their interactions in granulosa cells after long duration of growing phase of dominant follicles (7-day FSH) compared to conventional 4-day FSH treatment (reference group). Cells were obtained from the FSH-stimulated follicles 24 h after exogenous LH treatment in both groups. Genes involved in this network are markers of follicle LH responsiveness, oocyte nuclear maturation and oocyte competence. The differences in color intensity of molecules show the degree of up or down regulation. Network generated by Ingenuity Pathway Analysis software

In the network of differentially expressed granulosa cell genes (generated with IPA software; Fig. 3), most markers were those of LH responsiveness, oocyte nuclear maturation, and oocyte competence. The genes found to be up-regulated were: *regulator of G-protein signalling 2* (*RGS2*); *matrix metalloproteinase* (*Mmp*); *vanin* (*VNN2*); *guanosine triphosphate hydrolase* (*GTPase rho - Ras* (homolog); *peroxiredoxin 1* (*PRDX1*); *prostaglandin endoperoxide synthase 2* (*PTGS2*); *steroidogenic acute regulatory protein* (*STAR*); *mitogen activated protein kinase P38* (*P38 MAPK*); *mitogen activated protein kinase 14* (*MAPK14*); *phospholipase A2*, *group IVA* (*PLA2G4A*); *insulin-like growth factor 2* (*IGF2*); *selenoprotein P plasm 1* (*SEPP1*); *periostin osteoblast specific factor* (*POSTN*); *tumor necrosis factor, alpha-induced protein 6* (*TNFAIP6*); *pentraxin 3* (*PTX3*) and *plasminogen activator* (*PLAT*). The genes found to be down-regulated were: *inhibin beta B* (*INHBB*); *follistatin* (*FST*); *serpin peptidase inhibitor clade E member 2* (*SERPINE2*), *vascular endothelial growth factor C* (*VEGFC*), *inhibin beta A* (*INHBA*); *versican* (*VCAN*); *amphiregulin* (*AREG*); *low-density lipoprotein receptor related protein 8* (*LRP8*); *cytochrome P450 family 19 subfamily A polypeptide 1* (*CYP19A1*); *cyclin D2* (*CCND2*); *cyclin dependent kinase 1* (*CDK1*); *progesterone receptor* (*PGR*).

### Real-time PCR validation

**Fig. 4:**
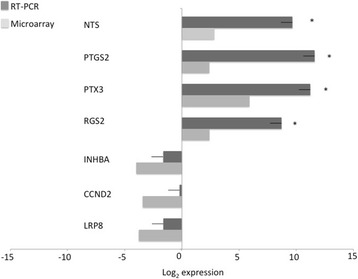
Quantification (log_2_ of fold-change; mean ± SEM) of the mRNA profiles in granulosa cells after a Long (7-day) compared to conventional (4-day, reference group) FSH treatment. Cells were obtained from the FSH-stimulated follicles 24 h after exogenous LH treatment in both groups. Bars extending to the right and left of zero represent up- and down-regulated genes, respectively, in the Long FSH group. RT-PCR data analysis was performed using REST 2009 program. Light grey bars represent expression of transcript in microarray experiment while dark grey bars represent expression of the same transcript obtained by RT-PCR. Asterisk (*) by the side of a bar represent statistical difference (*P* < 0.1) between long FSH in relation to the conventional FSH in RT-PCR experiments

Based on microarray data and functional analysis, 7 genes (*NTS, PTGS2, PTX3, RGS2, INHBA, CCND2* and *LRP8*) were selected for validation with RT-PCR. The selection of the genes for validation was based on the role they played in the results, as those genes were involved in the hypotheses being generated by the microarray analysis and not necessarily the genes with the most elevated fold change variations. The selected genes were quantified in three independent biological replicates (samples coming from three different animals) from the long- and conventional-FSH groups. The expression of 4 of 7 genes was statistically different between groups using RT-PCR (*NTS, PTGS2, PTX3* and *RGS2*) with a 90% confidence interval (*P* value ≤ 0.1), indicating a positive validation (Fig. 4). Although expression of the 3 remaining genes was not statistically different between groups (*INHBA, CCND2*, and *LRP8*), numerical differences followed the same trend as that of the microarray dataset. Since we have used a FDR filter, the chances of having false positive genes identified as differently expressed is lower although not impossible. The use of only 3 replicates was justified by the difficulties to generate more materials from such experiments. It is clear that, using more replicates may better support the PCR vs Array results where statistical power is increased by the measurement of 40,000 targets at the same time. The IPA analysis is especially made for such context and the analysis of tens of genes at the same time to assess a function or a pathway is more powerful in somatic tissues than individual PCR changes we can measure. This is the value of whole genome analysis.

### Follicular fluid analysis

Lower concentrations of estradiol were detected in the follicular fluid in the long FSH group than in the conventional FSH group (27.1 ± 7.6 vs 153.8 ± 32.7 ng/ml, mean ± SEM, *P* = 0.001). Conversely, follicular fluid progesterone concentrations were higher in the long FSH group than in the conventional FSH group (212.9 ± 24.5 vs 99.9 ± 19.7 ng/ml, P = 0.001). Accordingly, the estradiol:progesterone ratio was lower in the follicular fluid of the long- vs conventional-FSH group (0.13 ± 0.04 vs 3.5 ± 0.8, *P* = 0.0006).

## Discussion

The microarray technique provides a global analysis of the transcriptome of a tissue and is a hypothesis-generating tool. Microarray-based comparisons of mRNA transcripts of granulosa cells after 7-day FSH treatment versus the conventional 4-day treatment provide rationale for two postulates: Extending the superstimulation protocol by 3 days 1) activated molecular mechanisms of LH-responsiveness, and 2) increased expression of markers of oocyte competence in the granulosa cells. Therefore, follicles after long FSH were at optimal stage of differentiation to respond to an exogenous LH surge compared to follicles from the conventional FSH group. Taken together with our studies on superstimulation, oocyte maturation, ovulatory potential and embryonic development [16, 17, 25], results of the present study support the notion that 7-day superstimulatory treatment results in healthier follicles and more competent oocytes than the conventional 4-day protocol. Further, our results provide insights into the molecular mechanism for observed biological effects.

The LH surge is an important event that triggers resumption of meiosis in the oocyte and culminates in ovulation. At the molecular level, the LH surge initiates progressive changes in granulosa and theca cell functions including intercellular communications, tissue remodelling, steroidogenic and prostaglandin pathways, and mitosis. The specific genes influenced by the LH surge have been referred to as “markers of LH responsiveness” [33]. In our study, *VNN, POSTN, PLA2G4A, GTPase, Cysteine* (Fig. 3) were up-regulated in the long FSH group; these genes have been described as markers of the LH surge [33, 34]. Conversely, most of the down-regulated genes (*CYP19A1, LRP8, CJA1, INHBA* and *SERPINE2*) are those whose expression increase with dominant follicle growth till the time of LH surge (i.e., markers of pre LH surge) and are normally down-regulated after LH surge [35]. It is interesting to note that animals were ovariectomized 24 h after LH treatment; therefore, follicles in the long FSH group responded better to exogenous LH than those in the conventional FSH group. Although this is a really interesting finding, it may be too early to speculate a specific role of for some of those genes on granulosa cell maturation.

*Pentraxin 3* (*PTX3*) was the most up-regulated gene in the study. Previous studies document that its expression increased after the LH surge [36]. *PTX3* is also considered a marker of fertility [37, 38], suggesting that long FSH treatment may be associated with a more competent oocyte. In this regard, the effect of similar superstimulation protocols (long vs conventional FSH) on oocyte competence after in vitro fertilization were compared in a previous study [25] and although no morphologic differences were found between groups, long FSH treatment resulted in the production of 2.5 times more transferable embryos than the conventional FSH group [25]. In addition to the effect of LH on *pentraxin 3* expression, when endoplasmic reticulum stress was artificially activated in cumulus-oocyte complexes, the secretion of *pentraxin-3* was significantly reduced; in vitro fertilization rate was reduced, and embryos were slower to develop [37]. Perhaps a greater expression of *pentraxin* is the molecular explanation for greater embryonic development after a long FSH protocol in our earlier studies [25].

Prostaglandin biosynthesis in granulosa cells depends on the initial release of arachidonic acid from membrane phospholipids, and its production is increased by the LH surge [39]. Two of the up-regulated genes in the long FSH group were related to production of prostaglandin, *PLA2G4A* and *PTGS2*. Expression of *PLA2G4A* is responsible for LH-stimulated mobilization of arachidonic acid [40] via the adenyl cyclase/cAMP pathway [39], and expression of *PTGS2* (or *COX 2*) converts arachidonic acid to prostaglandin [41]. Inhibited expression of *PTGS2* has been associated with disturbances in ovulation [42–45]. Further, *PTGS2* was highly expressed in bovine preovulatory follicles after the endogenous LH surge [46], and has been implicated in oocyte maturation by differentially influencing multiple signaling pathways such as cAMP-dependent protein kinase, MAPK, NF-kappaB and phosphatidylinositol 3-kinase/AKT pathways [47]. Evidence of up-regulation of both enzymes (*PLA2G4A* and *PTGS2*) in the long FSH group in the present study is consistent with the concept that such treatment increased follicle maturity and ability to ovulate in response to LH than those after conventional FSH treatment. This concept is supported by the detection of a greater proportion of oocytes in metaphase II after a 7-day FSH protocol that a shorter conventional protocol [17].

Genes encoding the *low-density lipoprotein receptor-related protein 8* (*LRP8*), *cyclin D2* (*CCND2*) and *cytochrome P450 family51 subfamily A polypeptide1* (*CYP51A1*) were all down-regulated in the long FSH group in the present study, and have all been shown to be down-regulated after the LH surge [36, 48, 49]. *Cyclin D2* functions as a regulatory subunit of cyclin dependent kinase 4 or 6 (CDK 4 or 6) whose activity is required for cell cycle G1/S transition. *Cyclin D2* mRNA is present in granulosa cells of growing follicles, and its expression was induced by FSH but rapidly inhibited by LH [50, 51]. When ovulatory doses of human LH were administered to rats, *cyclin D2* mRNA and protein were rapidly decreased and were undetectable within 4 h. The presence of *CCND2* in human cumulus cells at the time of oocyte retrieval is also suggested to be a marker for lower embryo development and consequently low fertility [52]. Our finding that the *cyclin D2* gene was expressed at a higher level after conventional FSH treatment (i.e. down-regulated in long FSH group) demonstrate that extending the superstimulation protocol by 3 days may be beneficial to both follicular and oocyte health.

In an earlier study [24], we found that follicles superstimulated with a conventional 4-day FSH-protocol lagged behind in maturation and differentiation compared to the single (unstimulated) dominant follicle; i.e., most of the up-regulated genes were markers of the follicular growth phase [23]. In the present study, an additional 3 days of FSH stimulation resulted down-regulation of the same genes that had been up-regulated in the previous study using the shorter conventional FSH treatment, suggesting that the extra 3 days of gonadotropin support allowed follicles to reach a more mature, ovulation-ready state.

In cattle, intra-follicular aromatase activity increased during follicular growth [53] but decreased markedly by 24-h after LH [54, 55]. In the present study, the long FSH group had lower intrafollicular concentrations of estradiol and higher concentrations of progesterone than the shorter conventional FSH group. Although direct comparison between the 7-day FSH protocol and a single (unstimulated) dominant follicle was not possible in the present study, follicles in long FSH group were still estrogen active and not fully atretic, based on indirect comparison with follicular fluid concentrations of estradiol and progesterone in subordinate follicles [28]. Few apoptotic genes were up-regulated in the long FSH group in the present study; e.g., *Caspase 8*. Sequential activation of caspases plays a central role in cell apoptosis. Similar to the long FSH group, down-regulated genes in aged, persistent follicles included *amino acid transporter A2*, *aurora kinase family* (*A* and *B*), *fructose – 1,6 bisphosphatase* and *malate dehydrogenase* [56, 57]. Likewise, *glutathione S-transferase* isoform was up-regulated in both persistent follicles [56] and in the long FSH group in the present study. Early stages of follicle atresia have been associated with improved developmental capacity of oocytes [58–61]. Perhaps, changes analogous to the static or early regressing phases of a dominant follicle occurred in the long FSH group whereas follicles in the conventional FSH treatment group were still in the less differentiated growing phase.

## Conclusion

In conclusion, the extended protocol activates molecular mechanisms of LH-responsiveness and increases expression of markers of oocyte competence in the granulosa cells. Therefore, extending FSH treatment by 3 days allowed the granulosa cells to differentiate and proper respond to LH stimulus. These results support the notion that 7-day superstimulatory treatment is an alternative to obtain healthier follicles and oocytes.

## Abbreviations

aRNA: Anti-sense RNA
DNA: Deoxyribonucleic acid
ELMA: EmbryoGENE LIMS and microarray analysis
FDR: False discovery rate
FSH: Follicle stimulation hormone
IPA: Ingenuity pathways analysis
LH: Luteinizing hormone
LIMMA: Linear models for microarray analysis
LIMS: Laboratory information management system
PGF_2α_: Prostaglandin F2-alpha
RNA: Ribonucleic acid
RT-PCR: Reverse transcription polymerase chain reaction
